# Point-of-Care Testing—The Key in the Battle against SARS-CoV-2 Pandemic

**DOI:** 10.3390/mi12121464

**Published:** 2021-11-27

**Authors:** Florina Silvia Iliescu, Ana Maria Ionescu, Larisa Gogianu, Monica Simion, Violeta Dediu, Mariana Carmen Chifiriuc, Gratiela Gradisteanu Pircalabioru, Ciprian Iliescu

**Affiliations:** 1 National Institute for Research and Development in Microtechnologies, IMT-Bucharest, 077190 Bucharest, Romania; florina.iliescu@imt.ro (F.S.I.); ana-maria.ionescu@ucl.ac.uk (A.M.I.); larisa.gogianu@imt.ro (L.G.); monica.simion@imt.ro (M.S.); violeta.dediu@imt.ro (V.D.); 2 Department of Biochemical Engineering, University College London, Bernard Katz Building, London WC1E 6BT, UK; 3 Research Institute of University of Bucharest, University of Bucharest, 050095 Bucharest, Romania; carmen.chifiriuc@bio.unibuc.ro; 4 The Romanian Academy, 25, Calea Victoriei, Sector 1, 010071 Bucharest, Romania; 5 Academy of Romanian Scientists, 010071 Bucharest, Romania; 6 Faculty of Applied Chemistry and Material Science, University “Politehnica” of Bucharest, 011061 Bucharest, Romania

**Keywords:** SARS-CoV-2 variants, detection, epidemic, point of care testing

## Abstract

The deleterious effects of the coronavirus disease 2019 (COVID-19) pandemic urged the development of diagnostic tools to manage the spread of disease. Currently, the “gold standard” involves the use of quantitative real-time polymerase chain reaction (qRT-PCR) for SARS-CoV-2 detection. Even though it is sensitive, specific and applicable for large batches of samples, qRT-PCR is labour-intensive, time-consuming, requires trained personnel and is not available in remote settings. This review summarizes and compares the available strategies for COVID-19: serological testing, Point-of-Care Testing, nanotechnology-based approaches and biosensors. Last but not least, we address the advantages and limitations of these methods as well as perspectives in COVID-19 diagnostics. The effort is constantly focused on understanding the quickly changing landscape of available diagnostic testing of COVID-19 at the clinical levels and introducing reliable and rapid screening point of care testing. The last approach is key to aid the clinical decision-making process for infection control, enhancing an appropriate treatment strategy and prompt isolation of asymptomatic/mild cases. As a viable alternative, Point-of-Care Testing (POCT) is typically low-cost and user-friendly, hence harbouring tremendous potential for rapid COVID-19 diagnosis.

## 1. Introduction

Globally, at the end of November 2021, there have been more than 250 million confirmed cases of coronavirus disease 2019 (COVID-19), including five million deaths, reported to WHO. More than 1.5 billion vaccine doses have been administered so far [1]. Presently, the high transmission rate in communities and the mutagenic capability of SARS-CoV-2 contribute to the pandemic evolution and huge negative impact on health and economics [2,3,4,5]. The research community worldwide responded and gathered to consolidate the scientific findings and knowledge on SARS-CoV-2 infection to control the pandemic and prepare for future outbreaks. With the vaccination process and ongoing strict surveillance for side effects, this goal relies heavily on robust methods that combine early detection [6], isolation or physical distancing and therapeutic and epidemiologically-based prophylactic approaches. Clinical diagnosis of COVID-19 is possible when pathognomonic symptoms [7], coupled with epidemiologic data, are apparent. Since the clinical manifestations (pneumonia, dyspnoea, fever, cough, respiratory symptoms) are not always specific [8], and the history of contact with other possible infected persons is not always readily available [9,10], a rapid ascertainable clinical/epidemiological diagnosis is difficult. Therefore, laboratory procedures and data collected via effective screening and confirmatory diagnosis are crucial to identify COVID-19 asymptomatic/symptomatic cases, active or not.

Here, we review the recent advances in the detection of SARS-CoV-2. We will firstly present the main challenges in SARS-CoV-2 detection and a brief description of the virus biology. Further, we will discuss significant aspects of sample preparation. Main SARS-CoV-2 detection techniques such as nucleic acids amplification and testing, as well as serological testing, will be summarised. Finally, we conclude regarding the potential of Point-of-Care Testing (POCT) systems as rapid human and environmental testing methods.

## 2. Challenges in SARS-CoV-2 Detection

Consistent efforts have been concentrated on designing and manufacturing simple, rapid, accurate and affordable diagnostic kits to be used at home by anyone. Despite these efforts, the lack of diagnostic resources, human and technological, slowed down the progress and affected the outcomes of the protocols in place aimed at controlling the SARS-CoV-2 pandemic. Several technology-related aspects need to be considered when evaluating the situation for further improvements. For instance, it is known that most of the tests are portable benchtop analysers or even smaller handheld devices (i.e., MicrosensDx, RapiPrepCOVID-19, MesaBioTech Accula Test). However, all of these require sample preparation, which involves technical skills for viral transport media, pipetting, refrigeration and temperature re-equilibration. Moreover, limited access to reagents and equipment slows down the detection rate and hinders efforts to mitigate viral spread. The result is the relatively low throughput that imposes multiple processing units and dependence on the clinical setting for centralised testing. In the meantime, the clinical component is related to the immunological response, which takes time to be identified (the organism did not start developing antibodies against the virus) and varies from one patient to another (in some patients, the immune response is weak with untraceable antibodies), making the infection undetectable [11]. The clinical aspects, indeed, add to the technical difficulties. For example, one study [12] discussed the value of anosmia as a marker in mild cases excluded from testing. Furthermore, the study highlighted that the newly developed and persistent anosmia resulting from COVID-19 does not reflect these individuals’ infectious levels or the moment they have viral clearance. Other data also urged caution about smell tests as a screening tool in some settings, such as airports and shopping centres [13,14], despite the high positive predicting clinical value of new-onset and sudden-onset anosmia when the disease’s prevalence is high. Moreover, it is essential to consider that, if the virus mutates in the reverse transcription polymerase chain reaction (RT-PCR) target region, SARS-CoV-2 may not be detected or detected less predictably. Therefore, the performance characteristics of a test and its limitations in relationship with the epidemiology of the disease are imperative to avoid false-negatives (in case of high disease prevalence, inhibitors or medication interferences) and false-positives (when prevalence is moderate to low). The ideal combination is yet to emerge, as each test has its strengths and limitations. For instance, STOP (SHERLOCK Testing in One Pot) COVID [15] could be a valid aid in the “test-trace-isolate” type of test, especially in low-resource settings. However, the low throughput and the poor sensitivity of lateral flow-based systems at the early stages of SARS-CoV-2 infection hinder their use as POC molecular tests [16]. Similarly, 2020 data highlighted that the lateral flow devices for asymptomatic mass testing proved controversial due to their incapacity to detect the actual infection [17]. In the case of clustered regularly interspaced short palindromic repeats (CRISPR) [18], the major disadvantage is the lack of automation with manually operated protocols that increase sample contamination risk, despite being a highly reliable, specific and sensitive detection method [19]. The inability to test many patients resided initially in the limited biological specimens collected from suspect subjects. Currently, SARS-CoV-2 nucleic acid is generally detectable in saliva specimens during the acute phase of infection [20]. Therefore, the screening will continue to be evaluated and perfected as POCT in conjunction with patient history as well as clinical and other diagnostic information necessary to determine patients’ infection status. Also, information about the contaminated environment could be a significant element of the surveillance system during the COVID-19 pandemic. It has been observed that Ribonucleic acid (RNA) virus [21,22,23] is spilt in wastewater [24]. Therefore, standardised protocols are required for timely and accurate quantification of viral loads and a straightforward correlation with the community infection levels [25] and surveillance [26]. Also, detecting viral variants in wastewater could provide valuable information about the natural progress of viral epidemics and the-related contingency plan. Further work will improve the technology and results to increase the potential of existing methods as efficient detection and monitoring approaches.

## 3. SARS-CoV-2—Structure and Characteristics

Human Coronaviridae, a family of viruses with a positive-sense single-stranded RNA genome, includes seasonal viruses, such as HCoV-OC43, HCoV-229E, HCoV-HKU1 and HCoV-NL63 coronaviruses, which are known to circulate in the general population, being one of the most frequent etiological agents of seasonal respiratory tract infections (shCoV) [27]. In contrast, the severe acute respiratory syndrome coronavirus (SARS-CoV), Middle East respiratory syndrome coronavirus (MERS-CoV) and SARS-CoV-2 are highly pathogenic, causing life-threatening respiratory pathologies and having pandemic potential [28].

The SARS-CoV-2 virus is spherical with a rough diameter of 125 nm and club-shaped spike projections emanating from its surface, giving the virus its crown-like morphology (corona-virus) [29]. Infection is initiated by the specific binding of the spike protein (S) to the host cellular receptor angiotensin-converting enzyme 2 (ACE2). Importantly, ACE2 expression and tissue distribution are essential in viral tropism and pathogenicity. The SARS-CoV-2 spike protein interaction with host factors (ACE2 and the cell surface serine protease TMPRSS2) supports viral uptake and fusion with the cellular or endosomal membrane [30]. Inside the host cell, the coronavirus replicates its RNA genome, producing complete length copies that are subsequently incorporated into new viral particles released from the infected cell via exocytosis. The SARS-CoV-2 genome has around 27–32 kb, harbours 79% sequence identity with SARS-CoV and 50% with MERS-CoV [31] and is organised into six functional open reading frames (ORFs) [32]. The six functional ORFs encode for replicase (ORF1a/ORF1b), spike protein (S), envelope (E), membrane (M) and nucleocapsid (N). Seven putative ORFs encoding accessory proteins scattered among these structural genes have been identified [33]. A distinct trait of SARS-CoV-2 is the acquisition of a polybasic furin cleavage site (PRRAR) in the spike protein, which subsequently enhances its pathogenicity, zoonotic potential and transmissibility [34,35].

## 4. Specimen Collection and Sample Preparation

Diagnosis of SARS-CoV-2 infections can be performed on a variety of upper (throat, nasal, nasopharyngeal swabs/wash, saliva, sputum) and lower respiratory tract (bronchoalveolar lavage fluid) samples, as well as, although generally less reliably, on blood, urine and faeces. The laboratory diagnosis targets either the direct detection of viral antigens, nucleic acids or the host’s immune response (producing specific antibodies or T cells). However, the correct laboratory diagnosis of SARS-CoV-2 requires specimen collection (correct specimen at the right time) and preparation, regardless of the efficiency of the methods used [36]. Furthermore, despite being easy-to-collect and their relevance for resource-limited conditions, upper respiratory specimens might provide false-negative results in early infection cases, requiring repeated testing to increase sensitivity.

Sample preparation is critical, particularly for those specimens in transit from hospital settings towards POCT systems. Several difficulties were identified and related to maintaining the appropriate temperature until testing (refrigeration or freezing at −70 °C or below), obtaining the viral particles’ or antibodies’ appropriate concentration, reducing samples infectivity, overcoming the presence of inhibitors and decreasing the risk of sample cross-contamination and false-results. Such issues are particular to those diagnostic platforms involving separate reaction steps, tubes opening and liquid handling [37]. Therefore, various biofluids were considered when addressing these issues. For instance, self-collected saliva is an excellent non-invasive specimen for Point-of-Care diagnosis of SARS-CoV-2 infections. However, saliva samples contain enzymes (i.e., lysozyme), electrolytes, proteins, nucleic acids, hormones and indigenous microbiota, which might interfere with the diagnostic process. When using blood samples, most diagnostic tests require a separation step (plasma to serum). Moreover, only a small percentage of symptomatic hospitalised patients present detectable RNA levels in serum [7]. We will further highlight several aspects related to sample preparation specific to different SARS-CoV-2 diagnostic approaches.

### 4.1. Nucleic Acids

Viral nucleic acids (NA) can be isolated from saliva either directly or after the concentration of epithelial cells by centrifugation. The methods based on detecting viral NA are sensitive and safer because of the virions’ inactivation during preliminary sample purification steps. Viral RNA can be extracted using commercial kits, specific probe-conjugated magnetic beads after sample loading [38,39] or NaI-based binding solution (6 M NaI, 2% TritonX100, 10 mM HCl) coupled with silica particles (‘glass milk’) [39,40]. The Food and Drug Administration (FDA) authorised a few nucleic acid amplification-based methods as quantitative (Abbott ID NOW COVID-19, Xpert Xpress SARS-CoV-2) and qualitative (AcculaSARS-CoV-2 Test, Mesa Biotech Inc., San Diego, CA, USA) POCT. These comprise an automated, integrated RNA isolation step compared with other diagnostic systems (FastPlex Triplex SARS-CoV-2, Gnomegen COVID-19 RT-Digital PCR or Bio-Rad SARS-CoV-2 ddPCR Test) which need manual sample preparation [41]. Therefore, RNA extraction and purification are essential blockages in the development of POCT and research focused on removing these steps from the diagnostic workflow. Consequently, Alekseenco et al. [42] showed that SARS-CoV-2 infection could be successfully diagnosed on unextracted heat-inactivated nasopharyngeal samples using isothermal amplification. Joung et al. developed, in [15], STOP COVID (Specific High Sensitivity Enzymatic Reporter UnLOCKing Testing in One Pot), which does not need sample extraction and can be performed at a unique temperature with a single fluid handling step and one simple visual readout. Several other designs belonging to the group Real-time RT-PCR, Home Collection (i.e., GENETWORx COVID-19 Nasal Swab Test Kit [43], Verily COVID-19 RT-PCR Test [44], CRSP SARS-CoV-2 Real-time Reverse Transcriptase (RT)-PCR Diagnostic Assay [45], to “Saliva Collection Device” (TRUPCR SARS-CoV-2 Kit [46], SalivaDirect [20], Phosphorus COVID-19 RT-qPCR Test [47]), received Emergency Use Authorization (EUA) as self-collected at-home devices. Although rapid molecular assays showed variation in sensitivity (from 68% to 100%), this was lower compared to antigen tests (0–94%) [48].

### 4.2. Viral Antigens

SARS-CoV-2 antigen identification in saliva samples, buccopharyngeal swabs or nasopharyngeal aspirates using different methodological approaches (e.g., immunochromatographic fluorescence assay, quick-response lateral-flow) is highly dependent on virion density in the sample, which in turn depends on the host immune response. Notably, since the virion density in upper respiratory tract samples is low within a few days after the onset of symptoms, the risk of false-negative results increased. Therefore, the sensitivity of antigen tests has been shown to vary considerably across studies (from 0% to 94%) [48]. However, viral antigens are probably the most appropriate candidates for developing accurate, rapid, early and straightforward diagnosis methods. Therefore, novel approaches to concentrate the antigen or amplify the detection phase are needed to develop clinical applications.

However, serology tests, including enzyme-linked immunosorbent assays, chemiluminescence immunoassays and lateral flow assays, may confirm specific antibodies and a current or past SARS-CoV-2 infection. Deeks et al. [49] have reported significant differences in IgG, IgM, IgA and total antibodies sensitivity, with the lowest sensitivity recorded during the first week since the onset of the disease. The human saliva contains specific antibodies for viruses that are multiplying in the respiratory tract, such as influenza A, cytomegalovirus, SARS-CoV, Denga, Ebola, enteroviruses, EBV, mumps, HSV, measles, polio, rabies, rhino-, rubella, polyoma and hepatitis (VHA, VHB, VHC) [50]. Hence, saliva and throat or mouth swabs are non-invasive samples that can be used instead of blood for the serological diagnosis of different viral respiratory tract infections, including SARS-CoV-2 [51], allowing detection of both antigens or specific antibodies.

## 5. Detection Methods

### 5.1. Nucleic Acids Amplification Testing (NAAT)

**Figure 1:**
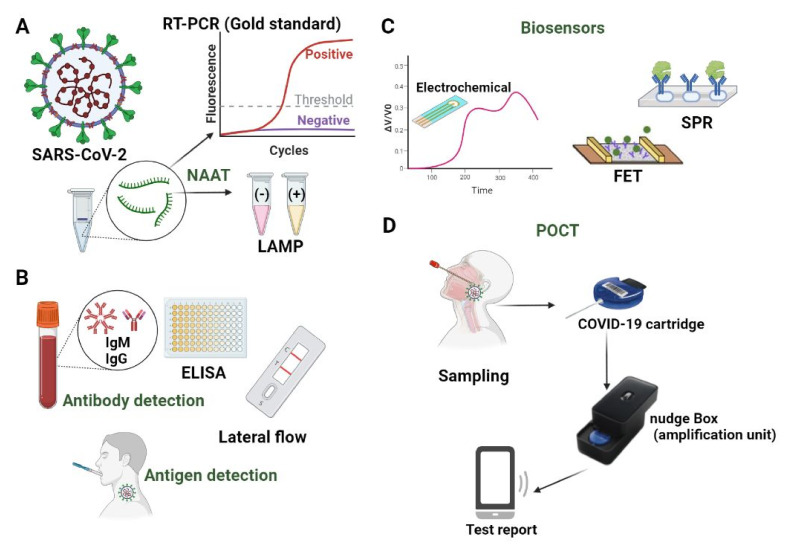
Examples of detection systems for SARS-CoV-2: (**A**) Nucleic Acids Amplification Testing-reverse transcription-polymerase chain reaction (NAAT-RT-PCR, gold standard for diagnostic), colorimetric real-time Loop-Mediated Isothermal Amplification (RT-LAMP) reaction; (**B**) detection of viral antigens and IgG/IgM antibodies using lateral flow tests/ELISA (enzyme-linked immunoassays); (**C**) schematic representation of a biosensor for SARS-CoV-2 detection; (**D**) schematic workflow of CovidNudge Point of Care diagnostic for SARS-CoV-2 (adapted from Gibani et al. [175]).

NAATs are diagnostic tests based on the amplifying of the RNA/DNA (Deoxyribonucleic acid) target to a point at which it becomes detectable. To date, COVID-19 is diagnosed using reverse transcriptase quantitative real-time PCR (RT-qPCR), and most of the FDA or FDA-Emergency Use Authorization (EUA) approved tests are real-time RT-qPCR. This technique employs fluorometric detection methods (probes or DNA intercalating dyes) and enables quantification and more straightforward automation than end-point PCR. However, the need for faster yet reliable testing for COVID-19 has pushed forward alternative NAATs to surpass the main drawbacks of RT-qPCR testing. The main alternative to PCR testing explores various isothermal amplifications, whereas a limited number employ sequencing-based technologies [52]. We will give a brief analysis of each of the techniques mentioned above. Figure 1 summarised the detection systems for SARS-CoV-2.

#### 5.1.1. PCR Mediated Detection

The publication of SARS-CoV-2’s first genome sequence in January 2020 [53,54,55] allowed several in-house and commercial molecular diagnostic kits to be developed and deployed globally (Xpert SARS-CoV-2, VitaPCR COVID-19 assay, RapiPrep COVID-19, ePlex SARS-CoV-2, Accula SARS-CoV-2, ID NOW COVID-9) as well as antibody-based tests (GT-100 SARS-CoV-2 IgG/IgM kit, rapid POC kit, COVID-19 IgM-IgG Rapid Test, COVID-19 Cassette, Rapid Test VivaDiag COVID-19 IgG-IgM test) [56]. These assays may run as standard real-time RT PCR (rt RT PCR)—thermocyclers or large automated or semi-automated diagnostic platforms on the respiratory sample (i.e., oro-/nasopharyngeal swab, sputum or bronchoalveolar lavage) sent to a reference laboratory for RT PCR testing. Since the turnaround time varies from 24 to 72 h, new approaches are required to improve the time-to molecular testing results. The Abbott’s m2000TM Real Time SARS-CoV-2 EUA test and the Roche’s Cobas^®^ SARS-CoV-2 Test, in hospitals and reference labs worldwide, are two examples. The FDA’s Emergency Use Authorized Cobas^®^ SARS-CoV-2 Test used the automated, high throughput Cobas^®^ 6800/8800 Systems, and up to 96 results were provided in about three hours. In eight hours, the Cobas^®^ 6800 System provided 384 results and, the Cobas^®^ 8800 System, 1056. The system’s specificity is assured by full process controls (negative, positive and internal) [57]. Furthermore, Aptima SARS-CoV-2/Flu assay, Hologic, Inc. is the first FDA-issued EUA automated multiplexed target nucleic acid amplification test intended for simultaneous in vitro qualitative detection and differentiation of RNA from SARS-CoV-2 virus, influenza A virus (Flu A) and influenza B virus (Flu B), isolated and purified from nasopharyngeal (NP), nasal and mid-turbinate swab specimens obtained from individuals suspected of respiratory viral infection consistent with COVID-19 by their healthcare provider. This combination is advantageous because it stemmed from a lack of testing devices during the flu season that coincides with the COVID-19 pandemic. Also, detecting SARS-CoV-2 nucleic acid in saliva specimens during the acute phase of infection is an advantage that can contribute to coherent screening via a rapid and accessible collection of biological specimens from COVID-19 suspects. Such screening via molecular laboratory-based assays that permit partial automation and may be faster might be recommended for urgent clinical cases (15–45 min) and will be evaluated and perfected as POCT in conjunction with patient history, as well as clinical and other diagnostic information necessary to determine patients’ infection status. POCT will be addressed in a separate chapter [58,59].

#### 5.1.2. Isothermal Amplification 

*Isothermal amplification* is carried out without thermal cycling and usually has a shorter turnaround time than PCR-based detection. Some isothermal NAATs, such as Loop-Mediated Isothermal Amplification (LAMP) [60,61] and Strand-Displacement Amplification (SDA) [62], amplify DNA targets and have a common principle: the enzyme complex is activated at approximately the same temperature as the primer annealing temperature. It performs both the denaturation of the double helix and the elongation of the synthesized strand, resulting in long (LAMP) or short (SDA and others) double stranded DNA (dsDNA) amplicons. Generally, they use a DNA polymerase with strand-displacement activity (i.e., Bst DNA polymerase), and by the addition of a reverse-transcriptase (RT) to the enzyme mix, DNA isothermal amplification can be adapted for RNA targets as well. In contrast, NA Sequence-Based Amplification (NASBA) [63] and Transcription-Mediated Amplification (TMA) [64] use an RNA polymerase (e.g., for phage T7) to synthesize ssRNA amplicons after the reverse-transcription (RT) of the RNA target. Various isothermal amplification reactions have been used for the detection of SARS-CoV-2 RNA, such as real-time LAMP(7) (RT-LAMP(7)) [65,66], Rolling-Circle Amplification (RCA) [67], Recombinase Polymerase Amplification (RPA) [68,69,70], NASBA [71] and TMA [72] in a tube or integrated on specialized detection platforms. The FDA-EUA list of isothermal NAATs revealed by RT-LAMP seems to be the preferred amplification strategy (see Table 1). Isothermal NAATs have the advantage of rendering faster results than PCR-based methods (under 30 min versus 2 to 4 h without an RNA extraction step). The assay time can be further reduced by shortening or skipping the RNA isolation step altogether. The evaluation of the effectiveness of RT-qPCR and isothermal NAATs provided different opinions regarding RNA extraction. For instance, [73] supported the importance of RNA extraction for an optimal assay, while [74,75] concluded that amplification from unprocessed biological samples or with minimum sample preparation (i.e., heat treatment) can also be an efficient detection strategy. Consequently, excluding RNA extraction from NAAT assays is a desirable procedural outcome because it reduces reagents, time, complexity and, implicitly, the costs of the test.

NAAT assays based on isothermal amplification can render end-point results or real-time readings and are compatible with different detection methods. For instance, RT-LAMP for SARS-CoV-2 diagnosis was developed with colourimetric [76] (Figure 1A), fluorescent [77] and CRISPR-based detection [19]. Unlike RT-qPCR, isothermal amplification assays are more likely to be optimized for qualitative results and cannot easily accommodate the quantitation of the target. Some attempts associated the colour change in RT-LAMP reactions with a determined initial concentration of the target. However, quantitation with isothermal amplification seems to only discriminate between very distant values [78].

Isothermal NAATs require means to ensure some minimal reaction conditions, such as the optimal ratio between reagents and sample, a working temperature and the integration of a suitable method for reading the assay results. However, they are more favourable for POCT integration than RT-qPCR in terms of complexity and costs. Moreover, several isothermal NAATs are currently available under FDA-EUA regulation, and most of them pave the road to genuine POCT by reducing the required laboratory equipment or even advancing user-friendly, over-the-counter devices for home testing.

LAMP-based alternatives to PCR emerged to overcome the time consuming and laborious detection by RT-qPCR-based techniques [61]. Indeed, the LAMP method is compatible with reverse transcriptase and was used for SARS-CoV testing on a benchtop assay [79]. This simple-to-perform technique uses four specially designed primers and amplifies DNA with high specificity, efficiency and rapidity under isothermal conditions (constant temperature of 65 °C). The technique’s efficacy may be improved by adding special fluorescent dyes or colour changing dyes to the reaction mixture. LAMP uses strand-displacement polymerases [80] rather than heat denaturation and provides a continuous amplification of RNA/DNA (up to 109 copies in less than 60 min). Since LAMP offers one-step detection (the sample preparation steps are simplified) and uses one single protocol, it also offers faster results. Moreover, it tolerates inhibitors and has higher stability [81] and sensitivity than PCR. A meta-analysis of NAATs on respiratory samples to detect coronaviruses shows that RT-LAMP assays have slightly lower sensitivity but are still comparable to RT-qPCR-based testing (75–90.5% vs. 78.1–98.5%) [82]. Since LAMP-based devices have been employed for viral detection, such as Avian influenza and human norovirus, it is a strong candidate for POCT in COVID-19. It detects the persistent disease and, thus, contributes significantly to active epidemiological surveillance. For instance, Abbott’s portable ID NOWTM Molecular platform offers healthcare providers the chance to perform molecular POCT outside the hospitals’ settings within minutes: positive results in as little as 5 min and negative results in 13 min. It tests the RdRp gene from the oro- and nasopharyngeal samples and is versatile and user-friendly [83,84]. Despite being cost-efficient, LAMP cannot provide information on previous infections with SARS-CoV-2, and none of the devices on the market have been designed as a use-at-home device by untrained people. Therefore, viable alternatives based on combinations of isothermal amplification and other methods were explored [85].

#### 5.1.3. Sequencing-Based Tests

Sequencing-based tests have also been developed for SARS-CoV-2 detection, employing either Sanger sequencing [86] or Next-Generation of Sequencing (NGS) technologies [87]. Although these tests are not as accessible as other NAATs, and require specialized personnel for data collection and interpretation, they are essential for surveillance testing of new viral variants. They offer information of utter importance for epidemic tracking, thus aiding pandemic management. However, the complexity of such tests currently rules them out as suitable candidates for integration on POCT devices.

#### 5.1.4. CRISPR-Mediated Detection

CRISPR-mediated detection is a biotechnological technique used for genome editing and is adapted to detect a specific NA sequence. It is considered to be a highly reliable, specific and sensitive detection method [18]. Several attempts investigated CRISPR for potential rapid COVID-19 testing and developed related protocols currently available online [88]. An essential advantage of CRISPR is the simplified detection process at a constant temperature of 37 °C. However, the major disadvantage is the lack of automation with manually operated protocols that increase sample contamination risk [89]. Moreover, it is essential to consider that, if the virus mutates in the RT-PCR target region, SARS-CoV-2 may not be detected or detected less predictably. Therefore, it is imperious to finalize the performance characteristics and evaluate its limitations in relationship with the epidemiology of the disease. For instance, the results provided by Sherlock Biosciences’ Sherlock^TM^ CRISPR SARS-CoV-2 kit are more likely false-negative when the disease’s prevalence is high and false-positives when prevalence is moderate to low. Moreover, inhibitors or other types of interference (i.e., common cold medications) may produce a false negative result. Therefore, further work will optimise the CRISPR-based detection method to suit the requirements of POCT devices.

#### 5.1.5. Combined Methods

Different types of isothermal amplification can be combined to obtain optimized NAAT assays. For instance, Penn-RAMP [90] synergistically combines RT-LAMP (specific due to the increased number of complementary primers per target) with RPA, which might be less specific but can increase the test’s sensitivity. One more example is the combination of LAMP and CRISPR-mediated detection: the SHERLOCK (Specific High sensitivity Enzymatic Reporter unLOCKing) technique, which offers reduced dependence on RT-qPCR equipment. The Sherlock^TM^ CRISPR SARS-CoV-2 kit by Sherlock Biosciences, Inc. employs one RT-LAMP-based, followed by one CRISPR-based, step. The RT-LAMP reverse-transcribes the targeted SARS-CoV-2 genomic RNA to DNA, and a strand-displacing DNA polymerase amplifies this. The subsequently amplified DNA transcription activates the collateral cleavage activity of a CRISPR complex programmed to the targeted RNA sequence. This cleavage of nucleic acid reporters makes possible a fluorescent readout detected by a plate reader. The confirmed limit of detection (LoD) for the Sherlock^TM^ CRISPR SARS-CoV-2 kit is 6.75 cp/mL VTM [91]. However, improvements are required to overcome the difficulties of fluid sample handling and to increase its potential as an outside-clinical-labs-testing device.

### 5.2. Serologic Tests

A significant component of the COVID-19 diagnostic and control is the serological testing to determine the prevalence of SARS-CoV-2 infection and measure the individual immune responses to SARS-CoV-2 infection or vaccination. Several studies have shown that convalescent-phase patient sera contain high SARS-CoV-2 spike-specific IgA, IgM and IgG antibodies with significant neutralising activity [92,93,94,95]. The spike protein’s sequence divergence from those of the widely circulating endemic hCoVs (30% sequence similarity of the S gene at the amino acid level) makes it an ideal antigen to detect and measure SARS-CoV-2 seroconversion. In September 2020, the U.S. FDA issued an emergency use authorisation (EUA) for the first serology (antibody) POCT for COVID-19. The Assure COVID-19 IgG/IgM Rapid Test Device was first authorised for emergency use by specific labs in July 2020 to help identify individuals with antibodies to SARS-CoV-2 and indicate ongoing or prior COVID-19 infection [96]. Also, the European Center for Disease Control and Prevention (ECDC) endorsed serologic tests for epidemiological and surveillance means only to monitor the viral status [97] and follow the immune response of affected subjects [55]. Interestingly, an Israeli national multi-centre task force validated, clinically and analytically, seven serology assays to determine their utility and limitations for SARS-CoV-2 diagnosis. Their results showed that ~5% of symptomatic SARS-CoV-2 positive patients remained seronegative across a wide range of antigens, isotypes and technologies [13]. It is acknowledged that, due to SARS-CoV-2 infection, the immune system responds and develops B lymphocytes able to secret specific antibodies, immunoglobulins (IgA, IgM and IgG). COVID-19’s natural progression and the kinetics of anti-SARS-CoV-2 antibodies revealed a 10–21-day window between the symptoms’ onset and the antibodies detection in serum [93,98]. Slight intra- and interpersonal variations for each type of Ig detected were identified, highlighting again the challenge in evaluating the antibodies’ effectiveness and understanding the virus’s pathogeny [99,100,101,102,103]. Detecting IgA in mild or asymptomatic forms of infection demonstrated one way to improve the diagnostic means while using blood and saliva samples [104]. IgM is the first immune response to the virus, while IgG has higher stability and persistency in serum, indicating infection stages. The specific antibodies, either attached to the B cells’ surface or free in the interstitial fluids, act as receptors for the viral antigens [105], more specifically the nucleocapsid [106] or spike proteins [94], and neutralise the virus’s effect [107]. The protective immune response of patients with COVID-19, precisely the IgG, decreases two–three months after infection [108]. Therefore, the main advantage of serological approaches is the identification of previous infections even without testing in the active phase of the disease. However, the main challenge is their insufficient accuracy, as the anti-SARS-CoV-2 immunoassays may present cross-reactions with other coronaviruses [109].

To date, several serological tests are considered, some of which were marketed as POCT. The available-on-the-market devices can be classified into:

1. Tests that detect a reaction and require trained personnel to interpret the results;
2. Tests that detect only the presence of antibodies by a colourimetric change.

The methods employed by serological tests include rapid diagnostic tests (RDTs), enzyme-linked immunoassays (ELISAs), chemiluminescent immunoassays (CLIAs) and neutralisation assays. Generally, RDTs, as POCT, rely on a cost-effective mobile non-automated method, most commonly lateral flow, to extract qualitative data in 5–20 min from a low sample volume without extensive specialised training. Despite these advantages, their accuracy is low, and their use is criticised as independent-from-the-centralised-laboratory types of tests. The lateral flow assay, ELISA and CLIA are more frequently used to test for IgG and IgM antibodies than a neutralisation assay that counts the neutralising antibodies that can effectively bind to and block virus replication. The clinical sensitivity and specificity of the existing commercial products are within 86%–93.5% and 96–100%, respectively. The majority of the rapid tests are paper-based devices manufactured worldwide (Table 2). Importantly, the FDA constantly updates the list of approved and distributed tests according to The Policy for Coronavirus Disease-2019 Tests [110].

The first rapid antibody blood test for SARS-CoV-2 developed by Cellex [111] and EUA approved by the FDA is a lateral flow immunoassay IgG/IgM SRT that provided 15–20 min results for total antibody. The clinical evaluation on blood, plasma and serum samples demonstrated a clinical sensitivity of 93.8% and specificity of 96.0%. Autobio Diagnostics Anti-SARS-CoV2 Rapid Test [112] and Chembio Diagnostic System’s DPP COVID-19 IgM/IgG system [113], also approved for EUA by the FDA, were lateral flow immunoassay tests to detect IgG and IgM. In comparison, Chembio Diagnostic System employed the DPP microreader for a qualitative readout to decrease the misinterpretations caused by visual detection of IgG/IgM. The clinical specificity was 97.6% for IgM, 96.8% for IgG and 94.4% for IgM and IgG combined. One example of a CLIA-based test, VITROS Immunodiagnostic Products Anti-SARS-CoV-2 Total Reagent Pack/Total Calibrator, detected total IgG/IgM in around 50 min, without differentiating them. The clinical sensitivity is 83% (30/36; 95% CI: 67.2–93.6%) and clinical specificity is 100% (400/400; 95% CI: 99.1–100.0%). La Roche modified the CLIA [114], as an electrochemiluminescent immunoassay (ECLIA), and developed Elecsys^®^ Anti-SARS-CoV-2 Test to detect, in only 18 min, the total antibodies against N protein. The clinical sensitivity is 100% and the clinical specificity is 99.81%.

Similarly, Bio-Rad’s Platelia SARS-CoV-2 Total Ab test by Bio-Rad Laboratories detected total antibodies against the N protein. Meanwhile, Abbott’s SARS-CoV-2 IgG Assay detected IgG against the N protein instead of total antibody levels. Table 2 presents the essential features of some of the FDA-EUA- and European Commission-approved serological tests: all tests can be used with serum or plasma samples as detection methods. The test’s sensitivity and specificity were evaluated in samples collected after a specific time interval (10–14 days) after symptoms onset of positive direct detection. Some tests are semi-quantitative or quantitative, ready to be used as POCT. The RDT commercially approved tests are paper-based biosensors for POCT.

**Table 2:** Main commercially available serological tests.

Test Name	Method/Technology	Manufacturer	Ig	Time [min]	Sensitivity/Specificity [%]	Reference
SARS-CoV-2 IgG Assay	Chemiluminescent microparticle immunoassay	Abbott Lab.	IgG only against N protein	~30	100/99.63	[115]
COVID-19 IgG/IgM Rapid Test Cassette	Immunoassay colloidal gold	Acro Biotech	IgM, IgG	~10	IgG 100/98 IgM 85/96	[116]
Anti-SARS-CoV-2 Rapid Test	Lateral flow immunoassay	Autobio Diagnostics	IgG and IgM only against S protein	~15	99.0/99.04	[117]
2019-nCoV IgG/IgM detection kit (colloidal gold)	Solid-phase immuno-chromatographic	Biolodics	IgM and IgG	~10	91.54/97.02	[118]
Platelia SARS-CoV-2 Total Ab assay	Semiquantitative ELISA	Bio-Rad Lab	IgA, IgM, IgG against N protein	~100	92.2/99.6	[119]
COVISURE™ COVID-19 IgM/IgG Rapid Test	Lateral flow immunoassay	W.H.P.M., Inc.	IgM/IgG	~15	IgM 76.7/97.1 IgG 70/97.1	[120]
qSARS-CoV-2 IgG/IgM Rapid Test	Lateral flow immunoassay	Cellex	IgG and IgM only against S and N proteins	15–20	93.8/96	[121]
Finecare TM 2019—nCoV Antobody Test	Lateral flow fluorescence immunoassay	Guanzhou Wondfo Biotech	IgM + IgG	~15	86.43/99.57	[122]
Clungene COVID-19 IgM/IgG rapid test cassette	Rapid immune antibody immunoassay test	Hangzhou Clongene Biotech	IgM, IgG	~15	87.1/98.89	[123]
LIAISON SARS-CoV-2 S1/S2 IgG	Chemiluminescent immunoassay	DiaSorin	IgG against S1/S2 protein	~35	97.56/99.3	[124]
Anti-SARS-CoV-2 ELISA IgG/IgA Anti-SARS-CoV-2 QuantiVac ELISA (IgG)/Anti-SARS-CoV-2 NCP ELISA IgG/IgM	ELISA for semi-quantitative and quantitative determination	Euroimmun (Perkin Elmer)	IgG, IgM, against S1 and nucleocapsid protein	15–60	94.4/99.6 IgA 100/92.4	[125]
VITROS Immunodiagnostic Products Anti-SARS-CoV-2 Total Reagent Pack	Chemiluminescent immunoassay	Ortho Clinical Diagnostics	Total antibody against S1	~50	100/100	[126]
COVID-19 IgG/IgM rapid test device	Lateral flow	RayBiotech			90.44/98.31	[127]
Elecsys Anti-SARS-CoV-2	Electrochemi-luminescence immunoassay	Roche	Total antibody against N protein	~18	100/99.81	[114]
Standard Q COVID-19 IgM/IgG Duo	Immunochromatography	SD Biosensor	IgM and IgG	~10	90.6/96.1	[128]
Atellica IM^®^ SARS-CoV-2 Total (COV2T)	Chemiluminescent microparticle immunoassay	Siemens Healthcare	Total antibody against RBD of S1 protein	~10	100/99.82	[129]
MAGLUMI 2019-nCoV IgM/IgG (CLIA)	Immune-antibody assay quantitative	SNIBE Co. Ltd.	IgM, IgG	~30	IgM 78.7/97.5 IgG 91.2/96	[130]
SGTi-flex COVID-19 IgM/IgG	Immunochromatography	Sugentech Inc.	IgM, IgG	~10	IgM 90.8/98.33 IgG 90.18/100	[131]
SGT Anti-SARS-CoV-2 Total Ab ELISA	ELISA		IgM, IgA, IgG	~150	Higher than Rapid test	

Presently, labs have used serologic tests to conduct major antibody seroprevalence studies and qualitatively analyse the previous exposure to SARS-C0V-2 [132]. Since the results are not for COVID-19 diagnosis, even when high IgM levels are observed, the serologic testing should be complemented by molecular testing to identify the RNA presence. Therefore, consolidating the diagnostic protocols and including clinical and lab evaluations [133,134] is imperative for timely identification of infection and exposure and vaccines’ efficacy evaluation. In this direction, Fluidigm launched the Community Connect Program to improve access to saliva-based COVID-19 testing. Furthermore, Thermo Fisher Scientific and the University of Oxford-developed Thermo Scientific OmnipathTM Combi SARS-CoV-2 IgG ELISA test detects and quantifies anti-SARS-CoV-2 antibodies and contributes to a higher university testing capacity (up to 50,000 tests per day). Furthermore, Euroimmun provided a complete package for COVID-19 diagnosis, which comprised FDA-EUA authorised PCR tests, differential serological tests and surrogate virus neutralisation tests [135]. Ongoing technological improvements such as the MAGLUMI^®^ SARS-CoV-2 diagnostic portfolio (MAGLUMI^®^SARS-CoV-2 S-RBD IgG, CLIA Kits, MAGLUMI^®^SARS-CoV-2 Neutralizing Antibody Assay) received the CE Mark recently to contribute and guide the clinical decisions in the combat of COVID-19 [136,137]. Similar outcomes would quantify the vaccines’ response accurately to assess the vaccine performance [138] and further develop immunodiagnostic tests for COVID-19 as cost-efficient, accurate qualitative and quantitative tests for POCT [3,139]. An integrated database analysis of available commercial tests was undergone to measure antigen and antibodies and which contributes to the scientific literature on COVID-19 tests methods and devices [41].

### 5.3. Lateral Flow-Based Detection

Lateral Flow Immunoassays (LFIA) or immunochromatography (LFIC) are currently the only available rapid POCT devices for SARS-CoV-2 diagnosis (Table 2). They detect SARS-CoV-2 antigens and related IgM and IgG. These tests provide short turnaround times (15 min) and do not rely on specialist laboratories or scientific analysis unless extensive batch testing is involved. This method is also cost-efficient, primarily when employed for serological tests. When used as molecular tests, the antigen assays detect the virus directly without RT-PCR or LAMP amplification steps. The testing devices generally comprise a shallow well and a porous test strip impregnated with antigens doped with a colourimetric indicator. Ten–fifteen μL of whole blood, serum or plasma are placed in the shallow well with each kit-specific buffer [93]. The buffer and the biological sample are to be absorbed in the porous strip. If the fluid sample is from a SARS-CoV-2 positive patient, the antibodies bind the immobilised antigens, triggering the colourimetric reaction. This antigen–antibody interaction leads to the formation of a narrow collar band, visible with the naked eye.

Joung et al. [15] developed a simple chemistry test suitable for POCT use, STOP (SHERLOCK Testing in One Pot). This simplified test, STOP COVID, designed to detect SARS-CoV-2 in one hour, provides sensitivity comparable to RT-qPCR-based SARS-CoV-2 tests and has a limit-of-detection of copies of viral genome input in saliva or nasopharyngeal swabs per reaction. The test returns the result in 70 min when using lateral readout and 40 min when using fluorescence readout. Moreover, it has been validated using nasopharyngeal swabs from COVID-19 patients and correctly diagnosed 12 positive and 5 negative patients of three replicates. The authors suggested that STOPCovid use would significantly support the “test-trace-isolate” initiatives, particularly in low-resource settings. However, the poor sensitivity of lateral flow-based systems at the early stages of SARS-CoV-2 infection and the low throughput hinder their use as POCT [16]. Recently, new data highlighted that the lateral flow devices for asymptomatic mass testing proved controversial. The report discussed the lacking performance as a POCT to “enable” a broadly distributed, affordable and rapid Innova lateral flow assay for repeat asymptomatic testing. The authors concluded that people testing negative must stick to infection control recommendations [17]. Furthermore, the main problem associated with this type of device is the incapacity to detect the actual infection. Since the immunological response takes time to be identified (the organism did not start forming antibodies against the virus) and varies from one patient to another (in some patients, the immune response is weak with untraceable antibodies), the infection may be undetectable [11]. Further work will improve the technology and results to increase this method’s potential to monitor post-infection and post-vaccination levels of immune responses efficiently. A detailed presentation of lateral flow devices for abroad diagnostics is summarized by Pohanka in [140] and Yadav et al. in [141].

### 5.4. Biosensors on Microfluidic Devices as POCT

Advancing biosensors-based POCT, either on-chip, paper or other materials, could be a solution for the rapid diagnosis of infectious diseases such as COVID-19. These methods, used to detect nucleic acids, antigens or antibodies in various unprocessed biological samples (saliva, sputum, blood), allow the readouts on colourimetric, fluorescent or electrochemical approaches. Most of the biosensors reported for SARS-CoV-2 detection are based on those developed for other types of genetic or biological sensors with an adjustment of the sensitive element targeting a specific viral protein or gene.

#### 5.4.1. Electrochemical Biosensors 

Electrochemical biosensors are the most versatile [142,143]. Electrochemical biosensors require simple instrumentation and they are highly sensitive, cost-effective and possibly miniaturised. Therefore, they are good candidates as Point-of-Care devices. Electrochemical biosensors usually employ the three-electrodes cell configuration: the working, the counter and the reference electrode. The working electrode, usually of gold, is modified with viral proteins, a probe complementary to the viral genome or antibodies. The surface modification gives the specificity to allow detection of viral antibodies, genomes or proteins. Results can be obtained using voltammetry techniques, such as amperometry, cyclic voltammetry (CV), differential pulse voltammetry (DPV) and square-wave voltammetry (SWV), as well as impedimetric techniques such as electrochemical impedance spectroscopy (EIS). Zhou et al. took a step forward and used electrochemical biosensors to detect various pathogens, including SARS-CoV-2. They reported an electrical detection of DNA hybridisation using non-Faradaic EIS [144], a sandwich-type electrochemical impedance immunosensor for Clostridium difficile toxin [145], and then a SARS-CoV-2 diagnostic method [146]. Furthermore, Rashed et al. [147] proposed a label-free EIS to detect SARS-CoV-2 antibodies using a commercial 16-well plate integrated with electrodes (from ACEA Biosciences). The electrodes were modified with a SARS-CoV-2 spike protein receptor-binding domain and tested with samples of anti-SARS-CoV-2 monoclonal antibody CR3022. The system was also tested on clinical serum samples. In the same direction, Chandra et al. [148] proposed a smartphone-assisted EIS platform using screen-printed carbon electrodes; however, there is no clear information about the testing of the device. An overview of electrochemical methods used for detection (including for SARS-CoV-2) is presented by Imran et al. in [149]. A portable molecular imprint polymer-based (MIP) electrochemical sensor for detecting the SARS-CoV-2 nucleoprotein through differential pulse voltammetry was reported by Raziq et al. in [150]. The functionality of the device was clinically tested on nasopharyngeal swabs. MIP biosensors have an excellent potential due to their long-term and thermal stability, cost and high specificity and stability; however, they present certain limitations related to the clustering of nanomaterials during synthesis. Nevertheless, the commercialization of MIP biosensors is still limited [151]. The potential electrochemical immunosensors for the fast testing of SARS-CoV-2 was analyzed by Ranjan et al. [152].

#### 5.4.2. Field-Effect Transistor (FET)

Field-effect transistor (FET) is another platform for the rapid and accurate detection of various analytes. Until now, it was used for detecting target analytes in gases and water. FET biosensors have a fast response and are relatively low-cost and easy to use. They can achieve high sensitivity and selectivity due to specific biomolecules immobilised conducting channels. This feature is considered a critical factor for FET sensor performance. Two-dimensional (2D) semiconductor materials, such as graphene, MoS_2_ and black phosphorous (BP), are mainly used as conducting channels due to their superior electronic properties. Reduced graphene oxide (rGO) was helpful as a conducting channel for detecting human immunoglobulin G20 [153]. The first report on using the FET sensor for detecting SARS-CoV-2 belongs to Seo et al. (Figure 1C) [154]. The conductive channel of graphene sheets was modified with a specific antibody against SARS-CoV-2 spike protein. The FET biosensor’s detection limit for clinical samples was established to be 2.42 × 10^2^ copies/mL. An overview of graphene-based biosensors for COVID 19 is presented by Sengupta et al. in [155]. Besides the lower cost of the device and its relatively simple testing setup, the main advantage of FET biosensors is that it does not require sample preparation or labelling.

#### 5.4.3. Plasmonic Biosensors

Plasmonic biosensors were used for label-free detection and are remarkably sensitive, fast and can give results in real-time. They are suitable for various types of clinically interesting target analytes where the main challenge is detecting small molecules at ultralow concentrations [156]. The special focus on this type of biosensor must be according to surface biofunctionalisation, which can potentially achieve the provision of an integrated lab-on-a-chip capable of transporting and detecting minutes of multiple bio-analytes with extremely high sensitivity and selectivity for dynamic monitoring at point-of-care levels. Functionalised substrates with antibody/antigen, aptamers and molecular imprinting offer specificity of detection [157]. A recent report described plasmonic biosensors for SARS-CoV-2 nucleocapsid antibodies detection used without diluted human serum [158]. Other work on COVID-19 lab diagnostics [159] presented two-dimensional gold nanoislands (AuNIs) functionalised with complementary DNA receptors, which can perform a sensitive detection of the selected sequences through nucleic acid hybridisation.

However, further work is required to develop the clinical sensitivity and specificity of the biosensors-based POCT for COVID-19 towards the levels of qRT-PCR ones (79–97% and 100%, respectively) [160,161].

### 5.5. Nanotechnological Approaches

Metallic, silica, polymeric or carbon-based nanomaterials (nanoparticles or nanotubes) functionalised with biomolecules such as DNA, RNA, peptides, aptamers, antibodies or antigens are used on a large scale for virus detection through colourimetric or antigen-binding assays [162,163,164,165] due to their optical, magnetic, electric or catalytic properties [166,167,168]. The main advantage of the nanomaterials is the high surface-to-volume ratio that can accelerate the sensor–analyte interaction for faster and effective detection of the virus [169]. Nanotechnological approaches were used in the detection of the SARS-CoV-2 virus. Moitra et al., in [170], proposed a colourimetric method based on plasmonic Au nanoparticles that were thiol-modified with antisense oligonucleotides and optimised specifically for the SARS-CoV-2 nucleocapsid phosphoprotein. In the presence of the targeted RNA, the gold-modified nanoparticles aggregated. The next step identified the positive samples if precipitation of Au nanoparticles occurred in the solution in the presence of RNase H. The processing time of the above-described method was relatively short (10 min), and the method did not require expensive instrumentation; however, the cost of the reagents limited its applicability. The graphene nanosheets of the FET [154] were modified with specific antibodies against the S protein. The device’s detection limit was 1 fg/mL S protein in buffered saline and 100 fg/mL in clinical transport medium. Nasopharyngeal swabs from patients with COVID-19 were used to validate the system, and the detection limit in clinical samples was evaluated at 2.42 × 10^2^ copies/mL. The method’s main advantage was that it did not require sample preparation, being also highly sensitive. Zhong et al. [171] used SARS-CoV-2 spike protein antibody-functionalised magnetic nanoparticles to detect SARS-CoV-2 by measuring the magnetic particle spectroscopy signal. Their study used 100 nm polystyrene nanoparticles conjugated with SARS-CoV-2 spike proteins for testing (no clinical samples). Detection of IgM and IgG antibodies is also based on gold colloidal nanoparticles in conjunction with lateral flow assay [99,172,173]. Chen et al. [174] reported the use of near-infrared (NIR) aggregation-induced emission nanoparticles for IgM and IgG detection using lateral flow immunoassay. The main advantage of the proposed method was the reduced autofluorescence of the nitrocellulose membrane when NIR excitation was used.

### 5.6. Point of Care Testing (POCT)

One way to control the COVID-19 outbreak that registered an exponential increase of cases in populations worldwide is to afford screening and diagnostic testing protocols that identify the positives quickly and accurately and facilitate quarantine and treatment. Since preanalytical factors contribute essentially to the testing procedure’s quality, the collection of specimens for on-site testing is crucial. In response to the SARS-CoV-2 pandemic, Gibani et al. assessed the performance of the redesigned DNANudge, UK into RT-PCR CovidNudge, as one rapid diagnostic test with no laboratory handling or sample pre-processing. The device, implemented in UK hospitals since May 2020, used naso- and oro-pharyngeal swabs and could facilitate rapid decisions for clinical care and testing programmes at a sensitivity of 93–94% and a specificity of 100%. The SARS-CoV-2 assay’s array consists of seven viral targets (rdrp1, rdrp2, e-gene, n-gene, n1, n2 and n3) and one control (Ribonuclease P, RNase P). It had a run time of less than 90 min and allowed safe testing outside a lab setting; however, the relatively low throughput imposed multiple processing units depending on the clinical setting for centralised testing (Figure 1D) [175]. The same principle used by laboratory-based testing permits partial automation and faster results (13–45 min). Therefore, they might be recommended for urgent clinical cases. However, the compromise between the turnaround time and the accuracy of the current systems is one serious barrier to be addressed. Since POCT is one viable alternative, it is supported by solid regulatory guidance for the laboratories that develop and implement COVID-19 molecular diagnostic tests. POCT provides an easy solution when mass testing is required because the testing protocol can be followed easily step-by-step, and the testing can be performed without any special medical training. Since the POCT systems are simple devices for easy-at-home use, they offer the users the ability to run a complete test, from sample preparation to results readouts, within minutes. Therefore, the users will seek timely and specific professional advice, which is essential for pandemic control.

Yin et al. [176] integrated multiplex digital RPA and fluorescence-based detection of nucleic acids into a sample-to-answer-chip-based PDMS device. The three-step process on a simple instrument and control system tested for nucleic acids in 45 min and proved to have immense potential as POCT in COVID-19 detection. Furthermore, automation and coupling with a smartphone are steps forward towards PoCT devices. For instance, Ma et al. [177] proposed a PDMS-LAMP-based system that quantified the results on colourimetric signals by HNB in 40 min. Most recently, an RT-LAMP-based benchtop assay demonstrated its performance and potential as a COVID-19 diagnosis tool: it similarly showed positive results with the WHO-approved RT-qPCR-based standard device [178]. Moreover, the LAMP protocol’s simplicity has been considered a great advantage for its use in POCT devices [161,179]. Such results further support the new protocol’s capability to be implemented on microdevices for at-home testing. The outcome will be effective for mass-at-home testing for a more robust prevention strategy for a diminished risk of spreading viruses such as SARS-CoV-2. More attractive than the chip-based alternative is the paper-based alternative, due to its cost-effectiveness. The method includes biodegradable materials and employs affordable fabrication processes that allow modifications and functionalization [180,181,182]. For instance, a lateral flow-based system can increase the potential of both molecular and serological testing for COVID-19 diagnosis. Since its first version in 2016 [183], several improvements have developed. One of them incorporated Fast technology Analysis (FTA) for nucleic acids extraction, LAMP for amplification (with small portable heater) and lateral flow for detection, all assembled with hydrophobic polyvinyl chloride to control the flow onto the platform [184]. Further simplification by Tang et al. [185] introduced semi-automation and on-board reagent storage and equipment free LAMP. The recent solution proposed by Reboud et al. [186] is a lateral flow-based test strip that incorporates elements to control the flow and prevent reagents from mixing. The new combination showed the ability to detect SARS-CoV-2 nucleic acids in less than 50 min in an unprocessed sample such as sputum. It also can be a future diagnostic tool in resource-limited locations. Trinh et al. [187] recently proposed two versions of 3D paper-based microfluidics sensors and evaluated molecular testing methods. The first is a fully integrated and foldable biosensor with LAMP-based amplification and multiplex fluorescence-based detection. The design included the reaction zone and the detection zone encapsulated with agarose for the reagents’ long-term storage. The detection used silver nitrate as a reaction indicator and UV light to visualise the reaction between the amplicons and the silver ions. The second model is an alternative that uses fuchsin for colourimetric detection of DNA amplicons. This version comprises a sample zone, a reaction zone (LAMP based process) and a detection zone that provide a simple naked-eye-detectable colourimetric signal. Furthermore, Kukhtin et al. [188] proposed a film-based biosensor as POCT of unprocessed samples via amplification and fluorescent imaging. The system is easy to fabricate, user-friendly, cheap and compatible with amplification processes. Moreover, it demonstrated its potential for detecting SARS-CoV-2 infection.

To date, there are commercial tests available to measure antigens and antibodies levels, but only a few are POCT [189]. The FDA-EUA-POC Xpert Xpress SARS-CoV-2 test by Cepheid, USA, provides results in 30 min using the GenXpert benchtop system. This simplified device is for medical offices and clinics. It is an automated, molecular RT-PCR test which qualitatively identifies SARS-CoV-2 multiple regions of the viral genome and Flu A, B and RSV in nasopharyngeal, nasal and aspirate samples. Cepheid’s Xpert Xpress Test detects both N2 and E SARS-CoV-2 genes, offering an additional assurance to the diagnosis [190]. Abbott Diagnostics introduced products closer to POCT concepts. The Scarborough, Inc.’s BinaxNOW COVID-19 Ag Card received the EUA for use at the POCT [191]. Interestingly, Abbott BinaxNOW COVID-19 Ag card’s evaluation as a rapid antigen diagnostic test at the Point-of-Care to supplement molecular testing was reported in a high-throughput, drive-through, free community testing site in Massachusetts [192]. The participants in the study, adults and children, were tested using paired reverse transcriptase PCR (RT-PCR)/BinaxNOW on anterior nasal (AN) swab samples upon providing their symptoms-related data. BinaxNOW demonstrated very high sensitivity in both age groups and high sensitivity in newly symptomatic adults, supporting the recommendations for using this test in adults with symptoms for less than seven days without RP-PCR confirmation. The study also highlighted the high throughput and the attention to be paid to fluctuations in temperature. Furthermore, the BinaxNOW COVID-19 Ag Card Home Test is a to-be-used-completely-at-home test. Interestingly, the test was offered by prescription in partnership with a telehealth service, bringing the POCT procedure to a new level: home collection and pool testing. However, caution was advised as the antigen tests are less sensitive than molecular PCR-based ones, even though highly specific. Compared with PCR, the FDA EUA ID NOW COVID-19 [193], as an isothermal nucleic acid amplification-based test, runs at a constant temperature, without thermal cycles, and amplifies six distinct regions on the target gene with the help of four sets of primers and a polymerase with high strand displacement activity. It targets one unique region of the RNA-dependent RNA polymerase (RdRP) gene of SARS-CoV-2. Therefore, the amplification is better and faster (~13 min for oropharyngeal, nasopharyngeal or nasal swab samples) than standard PCR. The kit is stable when stored at 2–30 °C and gives a simple method for mixing the sample with the specific reagents and transferring the prepared specimen for testing. The confirmed LoD in the natural nasopharyngeal swab matrix was 125 copies/mL, with analytical sensitivity and specificity of 100%. Clinical sensitivity and specificity were 100% at 2 × LoD and 5 × LoD, respectively. Similarly to Abbot’s test, Cue COVID-19 (Cue Health) [194] employed isothermal amplification with similar accuracy. This portable assay detected the SARS-CoV-2 N gene from nasal swab samples in less than 25 min and connected to a mobile phone to enable a diagnostic POCT platform.

For easy and fast diagnosis, multiplex platforms were considered. One example is Mesa Biotech Inc., which developed the Aculla SARS-CoV-2 Test as a multiplex that combines RT-PCR and lateral flow immunoassay and targets the SARS-CoV-2 N gene to detect the virus in nasal swabs specimens. The simple procedure, the visually displayed results and the short turnaround time (~30 min) indicate the test’s potential as POCT. The confirmed LoD in natural nasal swab matrix was 150 copies/mL, with an analytical sensitivity of 97%. The analytical specificity, Overall Precent Agreement (OPA), reported was 100% (95% CI: 93.15–100%) in a prospective clinical study, and 98% (95% CI: 89.35–99.95%) in a retrospective clinical study. Even though the accuracy of these systems is similar, the cost-effectiveness and user-friendliness levels favour the isothermal-based platforms. However, detecting genes on isothermal amplification-based devices is slightly more complex than on PCR-based systems, making these platforms preferred in clinical settings for diagnosis. Authorising multiplexed nucleic acids testing is one step towards simultaneous qualitative detection and identification of multiple respiratory pathogens. One example is BioFire Respiratory Panel 2.1 (RP2.1), in which the first COVID-19 diagnostic test granted marketing authorisation using the de novo review pathway. A multi-target POCT-based test recently received the marketing authorisation to screen individuals suspected of respiratory tract infections, including COVID-19, through testing for viral and bacterial nucleic acids in nasopharyngeal swabs [195]. The device supports the diagnosis of respiratory infection in conjunction with other clinical, epidemiologic and laboratory data or other risk factors. One factor that influences the efficiency of tests is the time factor eventually related to the collection of the specimens. This preanalytical step could be the key to fast and effective laboratory screening and timely diagnosis, depending on the collection timing and specimen types. MatMaCorp COVID-19 2SF Test (DBA MatmaCorp, Inc.) [196] and Real-time GENETWORx COVID-19 Nasal Swab Test and kit, RCA Laboratory Services LLC dba GENETWORx, received EUA limited to authorised laboratories for the qualitative detection of nucleic acid from SARS-CoV-2 in unsupervised at home self-collected nasal swab specimens when determined to be appropriate by healthcare providers. This test belongs to the group Real-time RT-PCR, Home Collection [197], similarly to Verily COVID-19 RT-PCR Test, Verily Life Sciences, one Real-time RT-PCR, Pooling, Home Collection [44] and CRSP SARS-CoV-2 Real-time Reverse Transcriptase (RT)-PCR Diagnostic Assay, Clinical Research Sequencing Platform (CRSP), for authorised laboratories at MIT and Harvard [45]. The rapid and accurate diagnostic strategy also included an ongoing evaluation of screening methods that use saliva specimens to determine patients’ infection status. Since the SARS-CoV-2 NA is generally detectable during the acute phase of infection, combining patient history and clinical and other diagnostic information increased the chances of successful POCT. Therefore, the FDA issued EUAs to special testing devices in the group called “Saliva Collection Device”. Some of the tests from this category are limited-to-authorised-laboratories tests and qualitatively detect nucleic acid from SARS-CoV-2 in saliva specimens collected with the assistance of a healthcare professional (HCP). For instance, TRUPCR SARS-CoV-2 Kit (3B Black Biotech India Ltd) incorporated the OMNIgene ORAL OM-505/OME-505 saliva collection device [46], while SalivaDirect (Yale School of Public Health, Department of Epidemiology of Microbial Diseases) permitted a fast and direct collection of saliva in sterile containers without preservatives [20]. One step forward was taken when combining Phosphorus COVID-19 RT-qPCR Test (Phosphorus Diagnostics LLC) and easy-to-use Oragene^®^ Dx (OGD-510) saliva collection was authorised as a self-collected at-home device [47].

The results of focused lab work on POCT for COVID-19 manifested as FDA authorisation of several tests for screening asymptomatic individuals as over-the-counter (OCT), with or without prescriptions, POCT [198]. This possible testing strategy is an essential step towards breaking the epidemiological chain of pandemic infection because it also reduces the exposure of medical personnel and identifies new cases in the population. Table 3 summarised the authorised POCT systems.

Other research attempts supported the role of various technological arrangements as POCT. For instance, the combination of electrochemical biosensors and the recombinase polymerase amplification (RPA) assay is a rapid, sensitive and convenient platform that can be potentially used as a Point-of-Care test to diagnose COVID-19 [208]. The combination worked without expensive thermo-cycling equipment and allowed the detection of multiple genes by differential pulse voltammetry, which was possible because of the hybridisation of the RPA amplicon with modified primers followed by amplicons’ build-up. The assay also demonstrated a better turnaround time and cost-efficiency compared with conventional PCR. The detection was better than the RPA based on electrophoresis without post-amplification purification: ~0.972 fg/µL for the RdRP gene and 3.925 fg/µL for the N gene.

The knowledge accumulated from studies on both analytical and clinical sensitivity of AgPOCTs, and compared with the standard RT-rtPCR, continue to support their utility as screening in outpatient departments and testing in the workplace or the general population. One recent study compared the performances of medical diagnostic devices available in many countries as outside-the-laboratory tests but restricted to the interpretation of results by medical personnel: Panbio COVID-19 Ag Rapid Test (Abbott, Jena, Germany), BIOCREDIT COVID-19 Ag (RapiGEN, St Ingbert, Germany), Coronavirus Ag Rapid Test Cassette (Swab; Healgen, Houston, TX, USA), COVID-19 Ag RespiStrip (Coris BioConcept, Gembloux, Belgium), RIDA QUICK SARS-CoV-2 Antigen (R-Biopharm, Darmstadt, Germany), NADAL COVID-19 Ag Test (nal von minden, Moers, Germany) and SARS-CoV Rapid Antigen Test (Roche-SD Biosensor, St Ingbert, Germany) [209]. These AgPOCTs have a short turnaround time and real potential on one condition: to have sufficient sensitivity and specificity. The conclusion of the study highlighted the limitations in sensitivity and specificity again. However, these results should be interpreted as (1) on-the-spot assessment of infectiousness and not as a concluded diagnosis in the very early and later phases of COVID-19; (2) including a need for confirmatory RT-rtPCR if possible; (3) a reminder that false-positives with AgPOCTs occur at a higher rate than with RT-rtPCR. With the sensitivity ranging within values that coincide with the infectious period in most patients, these assays (except BIOCREDIT COVID-19 Ag) could limit the virus transmission. Continuous studies are required for clinical validation accuracy confirmation of the AgPoCTs to incorporate them into clinical guidelines. Concomitantly, there is a need for a better understanding of serologic tests at the POCT level. For instance, the performance of a microfluidic quantitative immunomagnetic assay (IMA) (ViroTrack Sero COVID IgM + IgA/IgG Ab, Blusense Diagnostic) was compared with an enzyme-linked immunoabsorbent assay (ELISA) [210]. The results showed that, at 14 days after symptoms onset, the sensitivity of IMA was 91% (ELISA 91%) and specificity was 100% (ELISA 97.5%). Therefore, the study concluded that IMA for COVID-19 is a rapid simple-to-use POCT with accuracy similar to commercial ELISA. Since serological testing cannot replace RT-PCR for diagnosing acute COVID-19, it may serve as a valuable supplement when used to elucidate classic symptoms of COVID-19 associated with repeated negative RT-qPCR, while its primary application is to assess immunity. The FDA’s decision regarding the addition of the OTC and POCT for screening will give schools, workplaces, communities and others several options for serial screening tests that are accurate and reliable.

**Figure 2:**
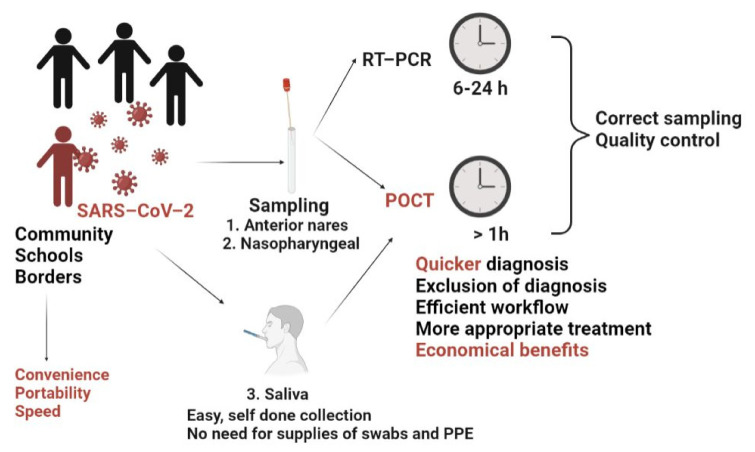
Point-of-Care Testing (POCT) advantages for the diagnosis and surveillance of SARS-CoV-2 infection.

It is very likely that the extensive use of POCT for COVID-19 virus testing paved the way for new models of healthcare delivery. Management of the SARS-CoV-2 pandemic has enabled a large number of stakeholders and patients to experience the benefits of POCT for the first time. POCT harbours several important advantages (Figure 2), including:

- clinical benefits (i.e., quicker diagnostic, exclusion of diagnostic, more appropriate treatment and subsequent improved clinical outcome);
- better access to testing in case of rural and remote sectors;
- economic benefits—POCT enables the rapid identification and isolation of infected individuals, hence avoiding lockdown measures.

## 6. Interpretating the Tests’ Results for Clinical Applications

Since COVID-19 quickly became a pandemic, new viral variants’ spread and the emergence require ubiquitous, fast, user-friendly, low cost and high-quality POC testing and results’ interpretation. In this context, mitigating the epidemiologic risk factors and relying on consistent guidelines (Table 4) for test specifications and requirements are crucial [211]. Therefore, applying mathematical relationships and visual logistics could help reveal patterns of SARS-CoV-2 tests’ performance [212]. Furthermore, established criteria (prevalence boundaries, predictive values, false omission rates, risk tolerance and repeat testing) of a realistic foundation for the design, selection and understanding of the disease diagnostics will diminish the risk for missed diagnosis [213]. Nevertheless, the difficulty in choosing the tests and the diagnostic protocols resides in interpreting and calibrating diagnostic tests’ performance. This task could be based on mathematical analysis that considers the above-mentioned criteria and compares positive predictive values (PPV) and negative predictive values (NPV) for the existing diagnostic test of COVID-19 (multiplex antigen, PCR kit, POCT antibody, home tests). [214] For instance, three performance tiers were derived for tests in relation to sensitivity, specificity, false omission rate and prevalence boundaries (tiered sensitivity/specificity comprises T1: 90%/95%; T2: 95%/97%; T3: 100%/>99%). Furthermore, graphical representations presented the uncertainty for EUA tests sensitivity and specificity (CI:95%) with regards to specific COVID-19 diagnostics and compared their mathematical Bayesian profiles at various prevalence levels. Visual logistics showed that, at a higher prevalence, false omissions are high, favouring the viral spread. Tier 3 (sensitivity 100%, specificity > 99%) offered a solution in a low prevalence setting. Furthermore, rapid antigen tests may assist in outbreaks with a large proportion of asymptomatic cases with high viral load (40%). The suboptimal sensitivity of the rapid antigen tests makes them less recommendable as a single test and requires a confirmatory molecular diagnostic test, especially in specific settings such as nursing homes and airports. Therefore, low prevalence is disruptive to all except the highest quality tests. Since prevalence is essential in clinical practice [215] and the SARS-CoV-2 pandemic develops with rapid emerging viral mutants, the prevalence is unpredictable, inconsistent and largely unknown [216], which imposes rapid testing and clear recommendations. For instance, multiplex molecular diagnostics (SARS-CoV-2, Influenza A/B, +/− RSV) are recommended in moderate prevalence settings as differential diagnostic tools. Conversely, the performance of rapid antigen test improves with prevalence and shows past infections. Furthermore, from a clinical point of view, single testing with low sensitivity rapid antigen tests (<90%) generates a high rate of false negatives as prevalence increases, hypothesising the safer-to-use repeat testing (e.g., rapid test followed by molecular diagnosis). Theoretical profiles show that repeat testing improves PPV and implicit test performance. Furthermore, the predictive value performance patterns suggest Tier 2 with PPA 95% and NPA 97.5% to optimize rapid testing [213]. The serological tests, which qualify under FDA EUA, require a sensitivity of 90% and a specificity of 95%. However, poor performance occurs at 80% prevalence due to increased false negatives relative to true negatives. Conversely, the ratio of false positives to true positives increases in low prevalence cases, and PPV decreased significantly. At an intermediate prevalence level (20%–50%), the risk of misdiagnosis is low. Therefore, one key to accurate diagnostics is the prevalence of the disease. Further detailed analysis of prevalence, mathematically derived calculations of PPV and NPV to reflect Bayesian (conditional probability) viewpoint of healthcare providers concerning tests’ performances, regulations (FDA, Infectious Disease IDSA) and clinical context will be required to ponder the value of positive test results and the merits of negative tests for COVID-19 for increased geospatial POC strategies.

## 7. Conclusions and Perspectives

Since the economic and psychological consequences of the COVID-19 pandemic are measurable at society and individual levels, the availability of specific and sensitive assays for at-home detection of this viral infection is essential. Medical professionals will gather information for accurate diagnosis, assess the outbreak’s extent and monitor intervention strategies and surveillance studies. Accurate and scalable POC tests to diagnose COVID-19 will increase the value of diagnosis made in the community and outside the lab settings while reducing the time to results, supporting rapid identification of COVID-19 patients and the appropriate isolation means, infection control measures, and enrolment into clinical trials of treatment. The current and evolving protocols [217] based on viral and antibody testing consolidate the clinical data for a clearer epidemiological picture. Currently, the primary targets of the diagnostic procedures are the viral genome (RNA) and the encoded proteins [218,219]. Different diagnostic tests have been developed for SARS-CoV-2 based on serological, molecular and nanotechnology techniques [93,219,220]. For instance, molecular testing is based on high throughput sequencing, reverse-transcription-polymerase chain reaction (RT-PCR), RT-loop-mediated isothermal amplification (RT-LAMP) and quantitative real-time PCR (qPCR) to detect the viral nucleic acid. Out of them, qPCR is recommended by the World Health Organisation (WHO) as the most rapid and effective method [66,85,221,222]. The range of molecular-based diagnostic tests has increased. However, they have severe limitations such as their unavailability in the remote and simple laboratory, primary or community settings, long turnaround times that facilitate nosocomial infections [223] or limited efficiency [224,225,226]. Therefore, serological testing was considered to be another critical component of the COVID-19 control effort. It is imperative to determine the prevalence of SARS-CoV-2 exposure and quantify individual immune responses to prior SARS-CoV-2 infection or vaccination. Available serological assays for SARS-CoV-2 used antigens derived from the spike or nucleocapsid proteins, the principal targets of the humoral response to natural infection [94]. Serological surveillance has become one crucial epidemiological tool during the COVID-19 pandemic, as it provides information regarding the protective antibodies and seroconversion after SARS-CoV-2 infection or vaccination and guides towards coherent patient care plans and public health policies [227].

Coupling clinical and laboratory data could identify active or past infection, as well as the individual at high risk of COVID-19, thus enabling prioritisation of PCR testing and quarantine and therapeutic efforts for absolute containment of the infection. However, a need for affordable and scalable tests to diagnose and monitor populations vulnerable to SARS-CoV-2 and gauge exposure at a population-wide level remains, especially when considering the asymptomatic individuals [228] and when shortcomings of molecular tests impede the rapid diagnostic and decisions. Thus, there is an urgent need for tests capable of providing accurate and timely qualitative and quantitative data, ideally from single sample measurements, which can be widely implemented [99,229,230]. It is already acknowledged that laboratories push their work performing molecular SARS-CoV-2 tests to increase their throughput and decrease the result rendering time.

Moreover, the efforts focus on an optimal testing protocol to use various clinical specimens to accurately detect SARS-CoV-2 infection by minimizing consumables usage and reducing hazard exposure to healthcare workers. In this review, we evaluated the diagnostic methods from various points of view, such as the type of test, the turnaround time, accuracy, adaptability, cost-effectiveness, setting’s capacity for specialised testing, the throughput and the integration of the recent developments. POCT may improve triage during the present pandemic and prepare for better community control, either as viral or surrogate tests [231,232]. Evaluating the current assays will support developing a self-consistent Point-of-Care system for the accurate detection of virus and infection progression testing and the global deployment of COVID-19 vaccines. Even if a test has high sensitivity and specificity (close to 100%), the prevalence of disease, user-to-user variation and sample type can impact the accuracy of a test. These values should be key measures that we use to diagnose and monitor COVID-19, beside testing frequency, vaccination rate and variant trends. Evaluating these key principles contributes to better assessment of tests’ performances and coherent testing strategies in risk reduction and disease management.

Recent advances in fabrication, manipulation and characterisation of nanomaterials should lead to the rapid and exciting development of new nanobiosensors, which can precisely and quickly detect minimal concentrations of analyte molecules even without pre-treatment or labelling [233,234]. Besides the established performances, nanobiosensors face several challenges which need to be addressed. One is to obtain nanoparticles/nanowires/nanotubes in a reproducible manner to ensure constant properties to permit scalability and reproducible nanobiosensors production for commercial usage. Different nanopatterning and functionalization technologies developed in the last two decades for biosensors development can also be used to obtain biosensors for COVID-19 diagnosis [154,235]. However, research needs to be intensified to develop antibody preparation techniques and for a better immobilization of immunological recognition elements on the surface of nanomaterials for performance improvement. Sensitivity and dynamic range should be matched to the patients’ SARS-CoV-2 viral load. Furthermore, nanobiosensors for COVID-19 detection require sample preparation (pre-concentration or pre-dilution) to match the analyte concentration with the dynamic range of the nanobiosensor, which increases the devices’ complexity and lowers its accuracy. The transport of analyte to the biosensor active surface, often provided by micro/nanofluidic devices, can increase the response time of the nanobiosensor. Therefore, new delivery systems were developed as solutions in patterned paper or evaporation-driven flow to reduce the complexity of the sensor device. Since nanomaterials facilitated the miniaturisation of the sensing platforms, new optimised fabrication techniques can further accelerate the transfer of this technology to commercial bionanosensors for COVID-19 detection. Finally, nanobiosensing should integrate the extraction system into the proposed biosensor as wearable and user-friendly e-skin devices. The present efforts in this rapidly developing domain should continue and materialise [236]. One possibility would be the microneedles-based biosensors to use interstitial fluid samples for continuous molecular and serologic monitoring of SARS-CoV-2 viral infection in symptomatic and asymptomatic populations. These wearable devices would provide important epidemiological and clinical information on the pandemic for better health policies and surveillance.

In conclusion, solving the existing or imminent significant challenges and investing in developing future IoT wearable nanobiosensors tailored to SARS-CoV-2 or other viral infections open new avenues towards rapid, accurate and in situ early diagnosis to track infectious diseases and eventually prevent further pandemic outbreaks. Extensive research synchronised with the healthcare industry goals will reach higher technological levels and propose sensitive and specific methods, and affordable, user-friendly, rapid, robust and simple diagnostic devices. The future belongs to comprehensive systems that integrate POCT and IoT for holistic approaches and “sample-to-answer” solutions [9,237].

## Figures and Tables

**Table 1:** Isothermal NAATs under Food and Drug Administration-Emergency Use Authorization (FDA-EUA, non-exhaustive). All data were collected from the documentation attached to each assay on the FDA website [41].

Assay & Company.	RNA Extraction	Isothermal Reaction	Type of Detection (LoD)	Required Platform
Color SARS-CoV-2 RT-LAMP Diagnostic Assay (Color Health, Inc.)	Require RNA extraction	RT-LAMP	Colourimetric (0.75 copies/μL)	Microplates read by spectrophotometry
Lucira CHECK-IT COVID-19 Test Kit (Lucira Health, Inc.)	Includes RNA extraction	RT-LAMP	Colourimetric (2700 copies/swab)	Microfluidic, over-the-counter device
Aptima SARS-CoV-2 assay (Hologic, Inc.)	Includes RNA extraction	TMA multiplex	Chemiluminiscence (0.1 TCID_50_/mL)	Automate system *Aptima Panther* (Hologic Inc.)
Cue COVID-19 Test for Home and Over The Counter (OTC) Use (Cue Health Inc.)	Does not require RNA extraction	unspecified	Electrochemical (20 copies/swab or 1.3 copies/μL)	Portable device *Cue Health Inc.*
Solana SARS-CoV-2 Assay (Quidel Corporation)	sample prep. (heat treatment)	RT-HDA	Fluorescence (1.16 × 10^4^ copies/mL)	*Solana* Instrument (Quidel Corporation)
Sherlock CRISPR SARS-CoV-2 Kit (Sherlock BioSciences, Inc.)	Require RNA extraction	RT-LAMP	Fluorescence aided by the enzymatic system CRISP/Cas (LoD = 6.75 copies/μL VTM)	BioTek NEO2 microplate reader
iAMP COVID-19 Detection Kit (Atila BioSystems, In.)	Does not require RNA extraction	OEMGA	Fluorescence (2000 copies/swab)	Real-time PCR instrument
MobileDetect Bio BCC19 (MD-Bio BCC19) Test Kit (Detectachem Inc.)	Does not require RNA extraction	RT-LAMP	Colourimetric (75 copies/μL)	In tube reaction.
ID NOW COVID-19 (Abbott Diagnostics Inc.)	sample prep. (heat treatment)	unspecified	Fluorescence (125 copies/mL)	ID NOW instrument
SARS-CoV-2 DETECTR Reagent Kit (Mammoth Biosciences, Inc.)	Require RNA extraction	RT-LAMP	Fluorescence aided by the enzymatic system CRISP/Cas (20 copies/μL VTM)	rtPCR instrument
ProAmpRT SARS-CoV-2 TEST (Pro-Lab Diagnostics)	Require RNA extraction	unspecified	Fluorescence (LoD = 125 copies/swab)	Genie HT Instrument (OptiGene)
SARS-CoV-2 RNA DETECTR Assay (USCF Health Clinical Laboratories)	Require RNA extraction	RT-LAMP	Fluorescence aided by the enzymatic system CRISP/Cas CRISPR/Cas (20 copies/μL)	Real-time PCR instrument

**Table 3:** Authorised POCT systems.

Name/Manufacturer	Type of Test	Detection/Turnaround Time	Sample	Intended Use	Performance	Sensitivity LoD	Reference
BD Veritor^TM^/Becton, Dickinson	Chromatographic digital immunoassay	Rapid test Qualitative det. Of viral nucleocapsid antigens 15 min	direct anterior nasal swabs from symptomatic, asymptomatic individuals for processing within 60 min	For prescription use as POCT/ screening with a prescription under CLIA with the BD Veritor™ Plus Analyzer	PPA: 84% (95% CI: 67%–93%) NPA: 100% (95% CI: 98%-100%)	1.4 × 10^2^ TCID50/mL	[189,199]
QuickVue At-Home OTC COVID-19 Test and Kit/Quidel Corp.	LFIA	Rapid test, Qualitative det. of viral nucleocapsid antigens 10 min	direct anterior nasal swabs	OTC at-home serial screening for symptomatic, asymptomatic individuals	PPA: 83% (95% CI: 74.9–89.6) NPA: 99.2% (95% CI: 97.2–99.8) does not differentiate between SARS-CoV and SARS-CoV-2	1.91 × 10^4^ TCID50/mL	[200]
BinaxNOW COVID-19 Antigen Self Test/Abbott Diagnostics	LFIC membrane assay	Qualitative detection of nucleocapsid protein antigen w/o viral transport media 15 min	direct anterior nasal swabs	OTC at-home serial screening for symptomatic, asymptomatic individuals	PPA: 77.2% (95% CI: 70.1–83.4) NPA: 98% (95% CI: 96.6–99.5) Sensitivity of the assay decreases over time. The presence of mupirocin may interfere with the BinaxNOW COVID-19 Antigen Self Test and may cause false negative results	140.6 TCID50/mL	[201]
BinaxNOW COVID-19 Ag Card 2 Home Test/Abbott Diagnostics	LFIC membrane assay	Qualitative detection of nucleocapsid protein antigen w/o viral transport media 15 min	direct anterior nasal swabs from symptomatic, asymptomatic individuals	OTC at-home serial screening only with the supervision of a telehealth proctor. The results are reported to the user and to the relevant public health authorities	PPA: 78.3% (95% CI: 71.1–84.4) NPA: 98% (95% CI: 96.6–99.5) Sensitivity of the assay decreases over time does not differentiate between SARS-CoV and SARSCoV-2.	140.6 TCID50/mL	[202]
BinaxNOW COVID-19 Ag 2 Card kit/Abbott Diagnostics	LFIC membrane assay	Qualitative detection of nucleocapsid protein antigen w/o viral transport media 15 min	direct anterior nasal swabs	POCT screening in patient care settings operating under a CLIA Certificate for symptomatic, asymptomatic individuals	PPA: 77.2% (95% CI: 70.1–84.4%) NPA: 98% (95% CI: 96.6–99.5%) sensitivity of the assay decreases over time does not differentiate between SARS-CoV and SARSCoV-2	140.6 TCID50/mL	[203]
MatMaCorp COVID-19 2SF Test/ DBA MatmaCorp,	RT-PCR + isothermal NAA	Qualitative detection of nucleic acid from SARS-CoV-2	nasopharyngeal, mid-turbinate, anterior nasal swabs	POCT screening in patient care settings. for symptomatic, asymptomatic individuals; it comprises sample preparation and amplification and detection.	PPA: 88.5% (95% CI: 79.5–93.8%) NPA: 100% (95% CI: 88.7–100%).	2000 genome equivalents per ml (100 genome equivalents per 50 µL reaction)	[204]
GENETWORx COVID-19 Nasal Swab Test and Kit/RCA Lab. Services LLC	rt RT-PCR (Home Collection)	Qualitative detection of nucleic acid from SARS-CoV-2	nasal swab	unsupervised at home self-collected samples, by qualified laboratory personnel	PPA: 99.6% NPA as expected with the correct collected samples	1.8 × 104 NDU/mL **	[43]
Verily COVID-19 RT-PCR Test and Kit/Verily Life Sciences	rt RT-PCR	qualitative detection of nucleic acid from SARS-CoV-2	anterior nasal, mid-turbinate, nasopharyngeal, and oropharyngeal swab	Pooling, Home Collection, unsupervised at home self-collected	PPA: 100% (95% CI: 89.9–100%) NPA: 100% (95% CI: 88.7–100%) low viral loads may not be detected in sample pools due to the decreased sensitivity of pooled testing	60 GCE/mL	[205]
CRSP SARS-CoV-2 RRT-PCR Diagnostic Assay/CRSP, LLC at MIT & Harvard	Real-time RT-PCR	qualitative detection of nucleic acid from SARS-CoV-2	unsupervised at home self-collected nasal swab	individuals suspected of COVID-19 by their healthcare provider	PPA: 98.3% (95% CI: 91–99.7%) NPA: 100.0% (95% CI: 96.6–100) Improper collection, transport, or storage of specimens may lower the efficiency	1600 copies/mL	[206]
Phosphorus COVID-19 RT-qPCR/Phosphorus Diagnostics	Real-time RT-PCR	the qualitative detection of nucleic acid from SARS-CoV-2 in	Upper respiratory tract swabs, washes/ aspirates, bronchoalveolar lavage (BAL) specimens from (2) saliva specimens	For individuals suspected of COVID-19 by their healthcare provider;	PPA: 95.0% (95% CI: 76.4–99.1) NPA: 99.2% (95% CI: 95.7–99.8)	5 copies/ µL in NP swab; 1.0 copy/µL in saliva	[207]

Legend: (PPA: Positive Percent Agreement = True Positives/(True Positives + False Negatives); NPA: Negative Percent Agreement = True Negatives/(True Negatives+ False Positives); ** NDU/mL: RNA NAAT detectable units/mL.

**Table 4:** The current IDSA recommendations for serology and molecular testing.

Test Type	Recommending for	Comments
Serological	the evaluation of patients with a high clinical suspicion for COVID-19 and with negative molecular testing at at least two weeks since symptom onset	The certainty of available evidence supporting the use of serology for either diagnosis or epidemiology was, however, graded as very low to moderate. [211]
	he assessment of multisystem inflammatory syndrome in children	
	the serosurveillance studies	
Molecular	all symptomatic individuals suspected of having COVID-19	prioritization of testing will depend on institutional-specific resources and the needs of different patient populations. [211]
	asymptomatic individuals with known or suspected contact with a COVID-19 case	
	asymptomatic individuals without known exposure is suggested when the results will impact isolation/quarantine/personal protective equipment (PPE) usage decisions, dictate eligibility for surgery, or inform solid organ or hematopoietic stem cell transplantation timing	
